# In Silico HCT116 Human Colon Cancer Cell-Based Models En Route to the Discovery of Lead-Like Anticancer Drugs

**DOI:** 10.3390/biom8030056

**Published:** 2018-07-17

**Authors:** Sara Cruz, Sofia E. Gomes, Pedro M. Borralho, Cecília M. P. Rodrigues, Susana P. Gaudêncio, Florbela Pereira

**Affiliations:** 1 LAQV-REQUIMTE, Department of Chemistry, Faculty of Science and Technology, Universidade NOVA de Lisboa, 2829-516 Caparica, Portugal; sm.cruz@campus.fct.unl.pt (S.C.); s.gaudencio@fct.unl.pt (S.P.G.); 2 Research Institute for Medicines (iMed.ULisboa), Faculty of Pharmacy, Universidade de Lisboa, 1964-003 Lisboa, Portugal; sestevaogomes@gmail.com (S.E.G.); borralhopm@gmail.com (P.M.B.); cmprodrigues@ff.ulisboa.pt (C.M.P.R.); 3 UCIBIO-REQUIMTE, Department of Chemistry and Department of Life Sciences, Faculty of Science and Technology, Universidade NOVA de Lisboa, 2829-516 Caparica, Portugal

**Keywords:** anticancer activity, HCT116 cell line, quantitative structure–activity relationship (QSAR), machine learning (ML), molecular descriptors, NMR descriptors, marine natural products (MNPs), marine-derived actinobacteria

## Abstract

To discover new inhibitors against the human colon carcinoma HCT116 cell line, two quantitative structure–activity relationship (QSAR) studies using molecular and nuclear magnetic resonance (NMR) descriptors were developed through exploration of machine learning techniques and using the value of half maximal inhibitory concentration (IC_50_). In the first approach, A, regression models were developed using a total of 7339 molecules that were extracted from the ChEMBL and ZINC databases and recent literature. The performance of the regression models was successfully evaluated by internal and external validations, the best model achieved R^2^ of 0.75 and 0.73 and root mean square error (RMSE) of 0.66 and 0.69 for the training and test sets, respectively. With the inherent time-consuming efforts of working with natural products (NPs), we conceived a new NP drug hit discovery strategy that consists in frontloading samples with 1D NMR descriptors to predict compounds with anticancer activity prior to bioactivity screening for NPs discovery, approach B. The NMR QSAR classification models were built using 1D NMR data (^1^H and ^13^C) as descriptors, from 50 crude extracts, 55 fractions and five pure compounds obtained from actinobacteria isolated from marine sediments collected off the Madeira Archipelago. The overall predictability accuracies of the best model exceeded 63% for both training and test sets.

## 1. Introduction

Colorectal cancer is the third most commonly detected cancer and the fourth foremost cause of cancer deaths in the world, accounting for about 1.4 million new cases and almost 700,000 deaths in 2012 [1,2]. The distribution of colorectal cancer burden varies widely, with more than two-thirds of all cases and almost 50% of all deaths occurring in more developed regions [2]. Therefore, colorectal cancer is considered one of the clearest markers of the cancer transition [1], replacing infection-related cancers in low developed regions that are undergoing rapid societal and economic changes together with other cancers predominantly linked to western lifestyles, which are often found in highly developed countries. Furthermore, drug research and development (R&D) is comprehensive, complex, expensive, time-consuming and full of risks process, i.e., the clinical success rate was only approximately 11% in 2014 [3].

Several new methodologies have been developed and applied in drug R&D to shorten the research cycle and to reduce costs. Computational methodologies have been instrumental at various stages of drug discovery [4,5], and continue to be indispensable in the incessant demand for life-saving drugs. Computer-aided drug design (CADD) methods have emerged as powerful tools in the development of therapeutically important small molecules for over three decades, with higher hit rates than that of the high throughput screening (HTS) approaches [6,7,8]. More than fourteen Food and Drug Administration (FDA)-approved drugs were mainly CADD-driven drugs [7,8,9], e.g., Imatinib (Gleevec^®^, Novartis, East Hanover, NJ, USA) a tyrosine-kinase inhibitor (anticancer approved drug, in 2001) that was developed using a multi-targeted drug design approach [8]. However, only few studies were reported on CADD for the inhibitory activity against HCT116 human colon carcinoma cells, which used small data sets that are generally focused on a single family of compounds e.g., flavonoid [10,11,12], pyrazole and furanopyrimidine [13], dispiroindoles [14,15], 2-pyrazolinyl-1-carbothiamide [16], *N*-acylbenzenesulfonamide [17], 6-chloro-1,1-dioxo-1,4,2-benzodithiazine [18], isosteviol [19], benzothiazole and pyrimido[2,1B]benzothiazole [20], 5,10,15,20-tetraaryl- and 5,15-diaryl-porphyrins [21], and platinum (IV) complexes [22] derivatives. Some studies built 3D quantitative structure–activity relationship (QSAR) approaches, by carrying out comparative molecular field analysis (CoMFA) and/or comparative molecular similarity indices analysis (CoMSIA), to design lead-like inhibitors against HCT116 cells [10,11,13,16,19]. Kim et al. [16] developed 3D-QSAR models, which were calculated using CoMFA and CoMSIA, for biological evaluation of thirty-seven 2-pyrazolinyl-1-carbothioamide derivatives against HCT116. The binding mode between the most active 2-pyrazolinyl-1-carbothioamide derivative and Abl 1, a tyrosine kinase, was elucidated by in silico docking [16]. Another study developed QSAR models using statistical learning techniques such as multiple linear regression (MLR) and molecular descriptors to modelling the anticancer activity against HCT116 cells [14,15,17,18,21,22]. Girgis et al. elaborated QSAR strategies to design spiro-alkaloids with anti-oncological activities using an MLR approach [14,15]. The most important descriptors for HCT116 models were LUMO (lowest energy unoccupied molecular orbital) for the 15 spiro[3*H*-indole-3,2′(1′*H*)-pyrrolo[3,4-*c*]pyrrole] derivatives [14] and minimum one-center core–electron attraction energy (plays an important role in steric and electronic parameters) and moment of inertia of mass (a geometrical descriptor) for the 24 spiropyrrolidinyl-oxindolyl derivatives [15]. Furthermore, molecular docking was also used to design lead-like inhibitors against HCT116 cells for flavanoid compounds on cyclin-dependent kinase-2 (CDK-2) target [12] and benzothiazole and pyrimido[2,1-*b*]benzothiazole derivatives on epidermal growth factor receptor tyrosine kinase (EGFR-TK) [20].

The declining number of new molecular entities (NMEs) in drug development pipelines together with higher success rate of drug discovery obtained from marine world (1 in 3500, marine natural products, MNPs [23]) as compared with the synthetic derivatives average (1 in 5000–10,000 compounds) have led to the rekindling of interest of NP-like scaffolds [24]. In spite of this, there is a need to develop new approaches to overcome the perceived disadvantages of new bioactive NPs discovery as compared with synthetic drugs, such as the difficulty in access and supply, the complexities of NPs chemistry, and the inherent slowness of working with NPs. There are numerous approaches to dereplication (i.e., the fast identification of previously elucidated NPs in an automated procedure), the most common are liquid chromatography with ultraviolet detection (LC–UV), liquid chromatography with mass spectrometry detection (LC–MS), liquid chromatography with tandem mass spectrometric (LC–MS/MS), and liquid chromatography with nuclear magnetic resonance detection (LC–NMR) providing structural information which is searchable in most of the commercial databases [25], and more recently MS/MS networking [26]. On the other side, access to 1D NMR data at the initial steps of dereplication of crude extracts can significantly accelerate the whole process [27]. Quinn and co-workers [28] reported a new NP discovery strategy that consists in frontloading of both extracts and fractions with desired physicochemical properties (i.e., LogP lower than 5, molecular weight (MW) lower than 500) prior to screening for malaria.

Here, we report the building of two QSAR studies using molecular and NMR descriptors for the prediction of anticancer activity against the human colon carcinoma HCT116 cell line, using the value of half maximal inhibitory concentration (IC_50_). In the first approach, A, regression models that predicted the IC_50_ value of anticancer activity were evaluated. These models were built using in total 7339 molecules that were extracted from the ChEMBL and ZINC databases and recent literature indexed in Web of Science. To mitigate NP discovery drawbacks of time consumption and biological activity screening-associated costs, we developed a new NP drug hit discovery strategy that consists of frontloading crude extracts, subsequent fractions and pure isolated compounds with 1D NMR descriptors that were used by the NMR QSAR models to predict compounds with anticancer activity prior to bioactivity screening for NP discovery, Approach B. In detail, the NMR QSAR classification models were built using 1D NMR data (^1^H and ^13^C) as descriptors, from 50 crude extracts, 55 fractions and five pure compounds obtain from actinobacteria isolated from marine sediments collected from the Madeira Archipelago [29]. The performance of the model was successfully evaluated by internal and external test set validations. Further external validations through data from more recent literature and using MNPs isolated in our research group were also accomplished.

## 2. Materials and Methods

### 2.1. Data Sets

#### 2.1.1. Approach A

In total 18,850 organic compounds were extracted from the ChEMBL (https://www.ebi.ac.uk/chembl/) [30] and ZINC (https://zinc15.docking.org/) [31] databases, searching by anticancer activity against the HCT116 cell line with IC_50_ values and their chemical structures saved in the simplified molecular input line entry specification (SMILES) data format. A search in the literature indexed in Web of Science Core Collection between May 2013 and October 2015 resulted in over 668 chemical structures which HCT116 activity records were reported. After collecting these databases, the duplicates were removed based on the IUPAC international chemical identifier (InChI) codes, however the chirality was taken into account, racemic compounds (or cases where no stereochemistry was indicated) were considered as one of the possible stereoisomers. For the duplicates with different IC_50_ values, the most recent were considered. After this, the final data set comprises 8958 compounds. A threshold of IC_50_ ≤ 10 μM (a cutoff for hit-to-lead anticancer activity studies) was defined after reviewing the literature [7,32,33] and searching National Institute of Health (NIH) for screening the NCI60 program (https://dtp.cancer.gov/discovery_development/nci-60/handling.htm). The IC_50_ values were converted to pIC_50_. The SMILES strings of the data set, the corresponding experimental and predicted activities are available as Supplementary Materials.

#### 2.1.2. Approach B

The data set comprises 50 crude extracts, 55 fractions and five pure compounds obtained from actinomycetes isolated from ocean sediments samples collected off the Madeira Archipelago [29], corresponding to 36 moderate-active-to-active (IC_50_ < 156 μg/mL) and 74 inactive (IC_50_ ≥ 156 μg/mL) samples against HCT116 cell line. Actinomycete strains were isolated from the marine sediments and the crude extracts were obtained through liquid–liquid extraction with ethyl acetate (EtOAc) in accordance with our previously reported work [29]. The EtOAc crude extracts were fractionated by silica flash chromatography, eluted with step gradients of isooctane/EtOAc followed by EtOAc/MeOH and were obtained nine fractions. Pure compounds were isolated by reversed phase HPLC (250 × 100 mm, 5 µm, 100 Å, 1.5 mL/min, UV 210, 250 and 360 nm, Phenomenex Luna, Torrance, CA, USA) using a gradient solvent system of acetonitrile and water. The code, type and the actinomycete genus of the samples comprising the data set, the corresponding experimental and predicted activity classes are available as Supplementary Materials.

### 2.2. Descriptors

#### 2.2.1. Approach A

JChem Standardizer tool version 5.7.13.0 (ChemAxon Ltd., Budapest, Hungary) was used to standardize the molecular structures by normalizing tautomeric and mesomeric groups and by removing small disconnected fragments. Three-dimensional models of the molecular structures were generated with CORINA version 2.4 (Molecular Networks GmbH, Erlangen, Germany), which did not provide results for a significant amount of compounds. Therefore, the data set was reduced to 7339 compounds. Empirical Molecular descriptors and fingerprints were calculated by PaDEL-Descriptor version 2.21 (http://www.yapcwsoft.com/dd/padeldescriptor/) [34]. Different types of fingerprints with different sizes were calculated and explored: 79 Estate (E-State fragments), 166 MACCS (MACCS keys), 307 Substructure (presence and count of SMARTS patterns for Laggner functional group classification—Sub and SubC respectively), 780 2D atom pairs (presence and count of atom pairs at various topological distances, AP2D and APC2D, respectively), 881 PubChem fingerprints (ftp://ftp.ncbi.nlm.nih.gov/pubchem/specifications/pubchem_fingerprints.txt), 1024 CDK (circular fingerprints), 1024 CDK extended (Ext circular fingerprints with additional bits describing ring features), 1024 CDK graph (specialized version of the FP which does not take bond orders into account), 4860 Klekotha–Roth (presence and count of chemical substructures, KR and KRC respectively) and a total of 1869 1D, 2D and 3D molecular descriptors (including electronic, topological, geometrical, constitutional and hybrid (BCUT, eigenvalues of a modified connectivity matrix, the Burden matrix, and WHIM, weighted holistic invariant molecular descriptors).

#### 2.2.2. Approach B

All samples were evaluated for HCT116 cytotoxic activity and the 1D NMR spectra were also acquired. NMR spectra were obtained using a Bruker Advance spectrometer, model ARX 400, (400 MHz for ^1^H and 100 MHz for ^13^C) with tetramethylsilane (TMS) as internal reference and deuterated chloroform as solvent. NMR spectra were handled with the ACD/NMR Processor (version 12.01, Advanced Chemistry Development, Toronto, Canada) and the range of chemical shifts used were 0–200 ppm and 0–12 ppm for the ^13^C and ^1^H, respectively. The NMR descriptors were generated using the following ranges: (1) 1.5 (133 descriptors), 1.0 (200 descriptors), and 0.5 ppm (400 descriptors) for ^13^C; and (2) 0.2 (61 descriptors), 0.1(120 descriptors) and 0.05 (240 descriptors) ppm for ^1^H.

### 2.3. Selection of Training and Test Sets

#### 2.3.1. Approach A

Two different approaches were used for the partition of the training and test sets. In one approach, the entire data set was divided into a training and a test sets of 5873 and 1466 compounds, respectively. The built QSAR models were developed and externally validated using the training and test sets, respectively. The approximate 4:1 partition for training and test sets, respectively, was carried out randomly according to the three categories of anticancer activity (i.e., active-to-very-active with IC_50_ < 10 µM, active-to-moderate-active with 10 µM ≤ IC_50_ < 50 µM, and inactive with IC_50_ ≥ 50 µM) in order to the biological diversity of the data set was captured by both sets. These three categories of activity were used only for the partition of the training and test sets. In the following experiments the definition of the limit of the anticancer activity against human colon carcinoma HCT116 cell line that was referred in Section 2.1.1 (active category with IC_50_ ≤ 10 μM) will always be used. In the other approach, the entire data set was divided into a training set of 5875 compounds and a test set of 1464 compounds, respectively. The approximate 4:1 partition was performed by a Kohonen Self-Organizing Map (SOM) [35] in such a way that both sets reflect the chemical diversity of the data set. The 7339 chemical structures of the whole data set were mapped on a SOM on the basis of Substructure fingerprint according to the three categories of anticancer activity. A tendency for clustering according to the structural classes of compounds was verified. The compounds for the test set were selected from occupied neurons and belonging to each of the structural clusters. The two approaches using the Substructure fingerprints were compared using the random forest (RF) algorithm in an out-of-bag (OOB) estimation, and a better performance was achieved by the SOM partition for the training set. The SOM partition between training and test sets of 5875 and 1464 compounds, respectively, was therefore included in the following experiments.

#### 2.3.2. Approach B

The whole data set, comprising 110 samples (50 crude extracts, 55 fractions, five pure compounds), was split into a training set of 74 samples (35 crude extracts and 39 fractions) and a test set of 36 samples (15 crude extracts, 16 fractions, five pure compounds), which were used for the development and external test validation of the QSAR models, respectively. The approximate 2:1 partition for training and test sets, respectively, was carried out randomly according to the two classes of anticancer activity (i.e., moderate-active-to-active with IC_50_ < 156 µg/mL, in total 34 samples, and inactive with IC_50_ ≥ 156 µg/mL, in total 76 samples) and the type of sample (i.e., crude extracts, fractions, or pure compounds) in order to the biological diversity of the data set was captured by both sets.

### 2.4. Selection of Descriptors and Optimization of QSAR Models

#### Approach A

The constant descriptors were removed and the training set was used to build MLRs [36] with Weka 3.7.12 [37] to select descriptors by the M5 method. With this method, all descriptors were used to build a first regression model, and then descriptors with the smallest standardized regression coefficients were removed in a stepwise way until no improvement was observed in the estimate of the average prediction error given by the Akaike information criterion (AIC) [36]. This procedure was separately applied to all the type of descriptors for a preliminary selection of descriptors. There is a demand for QSAR models with the minimum possible number of descriptors in order to develop more interpretable QSAR models, descriptor selection was further performed with the correlation-based feature selection (CFS) [38] algorithm implemented in Weka 3.7.12 (The University of Waikato, Hamilton 3216, New Zealand). The CFS takes into account the usefulness of individual descriptors for predicting the anticancer activity category or the pIC_50_ value together with the level of intercorrelation among them. The AttributeSelectedClassifier routine of Weka with the CfsSubsetEval option for evaluator and BestFirst, LinearForwardSelection, GreedyStepwise and PSOsearch options for search were used to compare experiments using the ten collections of descriptors, as well as different machine learning (ML) techniques. Selection of descriptors was accomplished using this procedure with the CFS algorithm within a ten-fold cross-validation methodology and *k*-nearest neighbors (*k*-NN) algorithm as ML technique. Optimization of QSAR regression models was performed using ten-fold or OOB cross-validation methodology with the training set employing the following statistical metrics: (1) R^2^, the square of the correlation coefficient (Pearson’s r); (2) RMSE, root mean square error; and (3) MAE, mean absolute error.

### 2.5. Machine Learning Techniques

#### 2.5.1. *k*-Nearest Neighbors

The *k-*NN technique predicts the activity category or the pIC_50_ value for a compound by majority voting of the *k* most similar compounds or by the average of the values for the *k* most similar compounds in the training set, respectively. Here, the *k*-NN algorithm was applied with the Weka (version 3.7.12) [37] using a *k* of 10, Euclidean distances, and contributions of neighbors weighted by the inverse of distance, which were optimized in ten-fold cross-validation methodology with the training set.

#### 2.5.2. Random Forests

A RF [39,40] is an ensemble of unpruned trees, which are created using bootstrap samples of the training set and for each individual tree the best split at each node is defined using a randomly selected subset of descriptors. A different training and validation set was used to create each individual tree. Prediction is made by a majority vote of the classification trees (classification) or by average of the individual regression trees (regression) in the forest. Moreover, the prediction error for the objects left out in the bootstrap procedure (internal cross-validation or OOB estimation) was used to assess the internal performance. The RF method quantifies also the importance of a descriptor by the increase in misclassification occurring when the values of the descriptor are randomly permuted, correlated with the mean decrease in accuracy parameter. RF give as well a probability to every prediction on the basis of the number of votes obtained by the predicted class. In the experiments presented here, RF were used for the development of classification or regression models to estimate anticancer activity against HCT116. The R program [41], version 3.2.3 was used to grow RF using the RandomForest library [42]. The number of trees in the forest was set to 500 and the other parameters, except mtry, were used with default values. The mtry parameter values were selected using factor levels of the default value (i.e., square root of the number of descriptors or 1/3 of the number of descriptors in the data for classification or regression, respectively).

#### 2.5.3. Support Vector Machines

Support vector machines (SVM) [43] map the data into a hyperspace through a nonlinear mapping (a boundary or hyperplane), for classification models the two class of compounds are separated in this space and for regression models a linear regression is performed in this space. In the current study, SVM models were explored with the Weka (version 3.7.12) [37] implementation of the LIBSVM software [44]. The *C*-SVM-classification or ε-SVM-regression types were chosen, the kernel function selected was the radial basis function and used the default value for the gamma parameter, and the parameter *C* was optimized in the range of 10−1000 through ten-fold cross-validation with the training set. The descriptors selected with the CFS algorithm within a ten-fold cross-validation for the training set were normalized and used to develop the classification and regression models.

### 2.6. Anticancer Screening in HCT116 Human Colon Carcinoma Cells

#### 2.6.1. Cell Culture

The HCT116 human colon carcinoma cell line was grown in McCoy’s 5A supplemented with 10% fetal bovine serum, 1% antibiotic/antimycotic (Invitrogen, Grand Island, NY, USA) and cultured at 37 °C in a humidified atmosphere of 5% CO_2_. The MTS metabolism assay was performed with cells seeded in 96-well plates at 3750 cells/well.

#### 2.6.2. Crude Extract and 5-FU Exposure

Stock solutions of 10 mg mL^−1^ of samples (actinomycete crude extracts, fractions, and pure compounds) and positive control 5-fluorouracil (5-FU) at 8 mM (Sigma, St. Louis, MO, USA) were prepared in dimethylsulfoxide (DMSO). Twenty-four hours after cell platting, cells were exposed to serial dilutions of samples and 5-FU, or DMSO vehicle control, for 72 h. All test samples, 5-FU or DMSO were serially diluted four-fold in culture medium.

#### 2.6.3. Evaluation of Cytotoxicity

To determine cancer cell response to chemotherapeutics and other compounds in targeted screenings, as well as to explore colon cancer signaling pathways, we tested the anticancer activity of samples in HCT116 cells. After 72 h of cell exposure to samples, the activity was evaluated and in parallel to the positive and vehicle controls. CellTiter 96^®^ aqueous non-radioactive cell proliferation assay (Promega, Madison, WI, USA) was used to anticancer activity evaluation using 4-(4,5-dimethylathiazol-2-yl)-5-(3carboxymethoxyphenyl)-2-(4-sulfophenyl), inner salt (MTS), according to the manufacturer‘s instructions. And the quantity of formazan product was measured after 1 h of incubation, using a Bio-Rad microplate reader Model 680 (BioRad, Hercules, CA, USA) at 490 nm. GraphPad Prism (version 5 GraphPad Software) was used to the IC_50_ values determination. All samples were diluted, resulting in final concentrations of the tested samples ranging from 156.2 to 0.08 µg mL^−1^.

## 3. Results and Discussion

**Figure 1:**
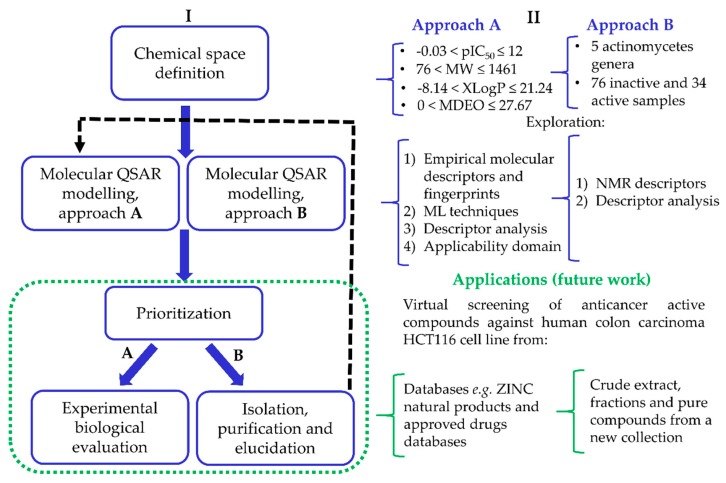
Flowchart representing the HCT116 quantitative structure–activity relationship (QSAR) model building process (**I**, left), illustrated with results obtained here as well as future applications (**II**, right).

In the current work, we report the building of two QSAR studies using the chemical structures of a data set of molecules and the 1D NMR spectra of a data set of samples (crude extracts, fractions and pure compounds) isolated from marine sediments collected from the Madeira Archipelago for the prediction of anticancer activity against human colon carcinoma HCT116 cell line, using the value of IC_50_. The two approaches, A and B, comprise several steps in order to build a comprehensive HCT116 QSAR model building process, Figure 1.

### 3.1. Chemical Space of the HCT116 Models

#### 3.1.1. Approach A

The whole data set (i.e., 7339 small molecules) was divided by the SOM into a training set of 5875 molecules (comprising 3441 active-to-very-active, Act-to-Vact, with IC_50_ < 10 µM, and 2434 inactive-to-moderate-active, Iact-to-Mact, with IC_50_ ≥ 10 µM, molecules) and a test set of 1464 molecules (comprising 1071 Act-to-Vact and 393 Iact-to-Mact molecules), which were used for the development and external validation of the QSAR regression models, respectively. The whole data set was clustered into 10 structural classes or scaffold types (I–X) using the ward tool in JChem. The ten structural clusters are represented in Table 1 along with as their average and maximum HCT116 pIC_50_ values.

Although, the molecules of these structural clusters are distributed over a wide range of pIC_50_ values between −0.03 and 12, all the ten clusters have an average pIC_50_ value higher or equal to 5.24 (corresponding to an IC_50_ value lower or equal to 5.75 μM), and a maximum pIC_50_ value higher than 9 (corresponding to an IC_50_ value lower than 0.001 μM).

The Lipinski rule only informs if a molecule is more likely to be an orally administrated active drug and if it is easily absorbed by the body. Furthermore, LogP is one of the most important molecular descriptors since it is highly correlated with lipophilicity, thus, more lipophilic molecules are often discontinued from drug development and are frequently related to toxicity issues [45]. Besides MW and LogP, we recently reported that the topological descriptor MDEO-12 (molecular distance edge between primary and secondary oxygen atoms) [46], the electronic descriptor TopoPSA (topologicalpolar surface area) [46] and the quantum-chemical descriptor HOMO (highest occupied molecular orbital energy) [47], have a remarkable performance in discriminating antitumor, antibiotic and overall biological lead-like compounds, respectively.

In order to exploit the training set chemical diversity, the Act-to-Vact and Iact-to-Mact molecules of the training set were analyzed, in accordance with the 10 structural clusters, using MW, XLogP (an estimation of the octanol-water partition coefficient, LogP) and MDEO-12. The analysis of these data indicates that the Act-to-Vact and Iact-to-Mact molecules against HCT116 in the training set are distributed over a wide range of MW (i.e., 76–1461 Da), XLogP (i.e., −8.14–21.24) and MDEO-12 (i.e., 0–27.67) values. The MDEO-12 descriptor is known to codify the molecular size by taking into account oxygen atoms also characterizes polarity [48] and provides an indication of the presence of oxygen-containing groups such as glycosyl, amide, lactam, ester or lactone together with hydroxyl, carboxylic acid or ether functional groups [46]. For example the Act-to-Vact spirostane-type saponin, orchidastroside A, from the Cluster I of the training set has the highest MDEO-12 descriptor value of 27.67. Interestingly, more than 63% of the compounds present in the training set have a MW that belongs to the interval between 300 and 500 Da. This MW interval contains approximately 64% and 62% of all Act-to-Vact and Iact-to-Mact molecules against HCT116 in the training set, respectively. However, using this rule (300 < MW ≤ 500 Da) it is only possible to discriminate Act-to-Vact molecules in relation to Iact-to-Mact molecules in three structural clusters, namely in Clusters III, VI and VIII, which comprises 79%, 82% and 77% of Act-to-Vact molecules as compared to 74%, 79% and 66% of Iact-to-Mact molecules, respectively. In addition, more than 66% and 74% of the Act-to-Vact and Iact-to-Mact molecules against HCT116 in the training set have an XLogP that is lower or equal to 5 and MDEO-12 that is lower than 0.999, respectively. Therefore, using the XLogP ≤ 5 and MDEO-12 < 0.999 rules it is possible to prioritize Act-to-Vact molecules in relation to Iact-to-Mact molecules in six (I, III, IV, VII, VIII, and X) and five (II, III, VIII, IX, X) structural clusters, respectively.

#### 3.1.2. Approach B

The whole data set, comprising 110 samples (50 crude extracts, 55 fractions, 5 pure compounds), was divided into a training set of 74 samples (35 crude extracts and 39 fractions) and a test set of 36 samples (15 crude extracts, 16 fractions, 5 pure compounds), which were used for the development and external validation of the QSAR classification models, respectively. Two classes of anticancer activity were set, moderate-active-to-active with IC_50_ < 156 µg/mL (in total 34 samples) and inactive with IC_50_ ≥ 156 µg/mL (in total 76 samples). The whole data set was divided into five actinomycetes genera (*Actinomadura*, *Brevibacterium*, *Micromonospora*, *Salinispora*, and *Streptomyces*) in accordance with our previously reported work [29]. The five actinomycetes genera are represented in Table 2 along with their activity classes and average HCT116 IC_50_ values.

It is interesting to highlight that the most abundant genera in our data set are *Streptomyces* (in total 50 samples, 36 and 14 samples in the training and test sets, respectively), *Salinispora* (in total 29 samples, 21 and 8 samples in the training and test sets, respectively), and *Micromonopora* (in total 27 samples, 15 and 12 samples in the training and test sets, respectively). The genus with the most bioactive potential against HCT116 is *Streptomyces*, comprising 20 (corresponding to 56%) and 7 (corresponding to 50%) active samples out of the 36 and 14 total samples in the training and test sets, respectively. It is not surprising since the genus *Streptomyces* over the last decades has stirred huge interest as a source of bioactive compounds, more than 60% of all known antibiotics have been isolated from streptomycetes [49].

### 3.2. Exploration of Empirical Molecular Descriptors and Fingerprints for QSAR Approach A

Two wide sets of descriptors were calculated by PaDEL-descriptor [34], one with 12 different types of fingerprints (FPs) with different sizes (79 Estate, E-State fragments; 166 MACCS, MACCS keys; 307 Substructure, presence and count Sub and SubC respectively; 780 2D atom pairs (presence and count of atom pairs at various topological distances, AP2D and APC2D, respectively), 881 PubChem fingerprints; 1024 CDK, circular fingerprints; 1024 CDK Ext, extended circular fingerprints with additional bits describing ring features; 1024 CDK graph, specialized version of the FP which does not take bond orders into account; and 4860 Klekotha–Roth, presence and count of chemical substructures, KR and KRC respectively) and other with a total of 1869 1D, 2D and 3D molecular descriptors (including electronic, topological, geometrical, constitutional and hybrid, BCUT and WHIM, descriptors). For the calculation of the 3D molecular descriptors the 3D models of the molecular structures were generated with CORINA. The performances of the two sets of descriptors in QSAR experiments in predicting pIC_50_ against HCT116 were compared. These exploratory QSAR experiments employed selection of descriptors with the CFS filter [38] followed by the simple k-nearest neighbor (*k*-NN) prediction of pIC_50_ against HCT116, within a ten-fold cross-validation procedure (Table 3).

The CFS filter maximizes the correlation with the variable to predict and minimizes intercorrelation between descriptors. The two molecular descriptors sets,1D2D and 1D2D3D, and the four fingerprints sets, CDK, CDK Ext and PubChem, achieved the best results, taking into account the value of the RMSE (Table 3, the best models are highlighted in bold). From the fourteen sets of descriptors and fingerprints, only 1D2D, 1D2D3D, CDK Ext, and PubChem fingerprints were used in further investigations.

#### 3.2.1. Exploration of Other State-of-the-Art Machine Learning Techniques

A comparison of three ML techniques, RF, SVM, and *k*-NN, for building QSAR models with the descriptors are described in Table 3 for SVM and *k*-NN, and without selection for RF is shown in Table 4.

**Figure 2:**
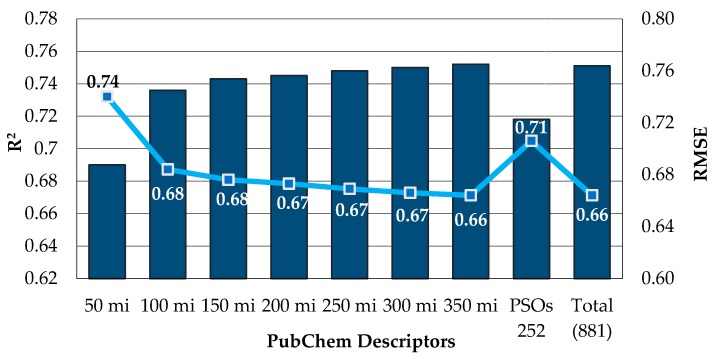
Analysis of Descriptor Selection Using RF algorithm in an OOB estimation for the training set.

In general, we did not observe an effective improvement in performance of RF algorithm with descriptor selection as has been reported in literature [39]. Here, RF showed a better performance when compared to SVM and *k*-NN for all descriptors sets (i.e., 1D2D, 1D2D3D, PubChem, CDK) in the prediction of the pIC_50_ against HCT116 taking into account the value of the RMSE (Table 4). The best model was accomplished by the RF using the PubChem fingerprints for the training set with an R^2^ of 0.751 and RMSE of 0.664. This model was further optimized through descriptor selection, based on the importance assigned by the RF model—Figure 2.

The selection of the 350 most important (mi) descriptors from the PubChem fingerprints set used to build the model with the RF enabled the training of much smaller RF models with even better prediction accuracies (R^2^ = 0.752, RMSE = 0.664 and R^2^ = 0.729, RMSE = 0.689) than the models trained with the whole set of descriptors (881 descriptors) for the training and test sets, respectively. The analysis of the best HCT116 QSAR model by the ten structural clusters, was displayed in Table 5 for training and test sets. In general the predictions obtained for the structural clusters are better than those obtained for all training set taking into account the RMSE value, except for Clusters I, IV, and VIII (bold highlighted in Table 5).

**Figure 3:**
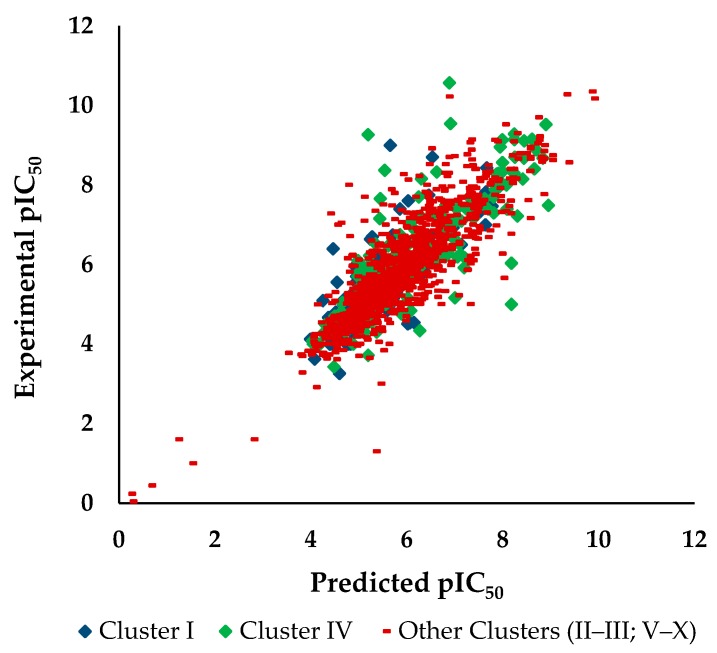
Predicted vs. experimental pIC_50_ against HCT116 for the 129, 200 and 1135 molecular structures of I, IV and others clusters of the test set, respectively.

Interesting, the worse prediction obtained taking into account the RMSE value for all the ten clusters in the test set was also to the Cluster IV. Analyzing the number of outliers, i.e., with an absolute error greater than 3 × MAE (0.455), in each of the ten clusters of the test set allows to identify two clusters (I and IV) that stand out for having a percentage higher than the one obtained for all the test set (78 outliers, 5.33%), 7.75% and 7.50%, respectively. Figure 3 represents the plot of predicted vs. experimental pIC_50_ values against HCT116.

An improvement in the RF model prediction accuracies (R^2^ = 0.742, RMSE = 0.665) was achieved for the other clusters (Clusters II–III and V–X) of the test set as compared with the prediction accuracy obtained for all the molecules of the test set (R^2^ = 0.729, RMSE = 0.689). For the Clusters I and IV inferior prediction accuracies were obtained: R^2^ = 0.613, RMSE = 0.673 and R^2^ = 0.682, RMSE = 0.821 respectively.

#### 3.2.2. Analysis of PubChem Fingerprints Identified as Relevant for Modeling the pIC_50_ Against HCT116

The PubChem fingerprints (FPs) comprise 881 FPs that can be divided into seven section types i.e., Section 1—Hierarchic Element Counts (HEC), 145 FPs; Section 2—Rings in a canonic Extended Smallest Set of Smallest Rings (ESSSR), 118 FPs; Section 3—Simple atom pairs (SAP), 64 FPs; Section 4—Simple atom nearest neighbors (SANN), 89 FPs; Section 5—Detailed atom neighborhoods (DANh), 44 FPs; Section 6—Simple SMARTS patterns (SSP), 253 FPs; and Section 7—Complex SMARTS patterns (CSP), 168 FPs. The twenty most important PubChem FPs, found by the best RF model, combined two HEC FPs, two ESSSR FPs, two SAP FPs, four SANN FPs, one DANh FP, six SSP FPs, and three CSP FPs. In Table 6 we describe the twenty most important PubChem FPs for modeling the pIC_50_ against HCT116.

In the set of the twenty most important descriptors, the descriptors codifying the presence of oxygen-containing groups are very relevant; eleven descriptors out of twenty most important descriptors. The alcohol functional group appears to be very relevant for modeling the activity against HCT116 and is codified by the descriptors CSP_819 (the 2nd), SANN_339 (the 11th), SSP_631 (the 20th) and may be also codified by the descriptor SANN_346 (the 15th). The others oxygen containing groups were methyl ketones (DANh_432, 8th), hydroxylamines (SSP_514, 12th), alkoxy alkylamines (SSP_615.13th), α,β-unsaturated carbonyls (SSP_672, 14th), nitro or *N*-oxide groups (SAP_301, 18th) and may be also codified aldehydes (SANN_346, 15th). Moreover, the methyl group appears also to be very important and is codified by several descriptors, namely the dimethylphenyl group (para-substituted, CSP_713, the 1st and ortho-substituted, CSP_755, the 9th), and 2-methylcyclohexanol (CSP_819, 2nd).

#### 3.2.3. Applicability Domain of the pIC_50_ Against HCT116 Model

**Figure 4:**
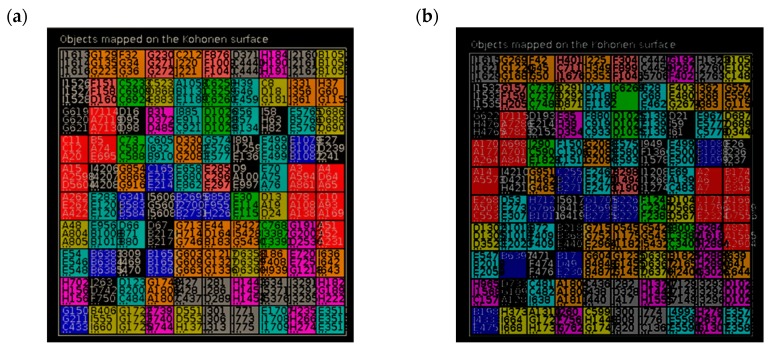
Mapping of the trained and predicted structural clusters of the active and inactive molecules against HCT116 on SOM for the: (**a**) training set; (**b**) test set. Red—Cluster I, Dark blue—Cluster II, Green—Cluster III, Light yellow—Cluster IV, Light blue—Cluster V, Pink—Cluster VI, Dark yellow—Cluster VII, Purple—Cluster VIII, Dark grey—Cluster IX, Light grey—Cluster X.

A definition of applicability domain based on the similarity between a molecule of an external data set and all the 5875 molecules in the training set was explored. The molecules of the training set were mapped on a Kohonen self-organizing map (SOM), using in-house developed software based on JATOON Java applets [35], on the basis of the 307 Substructure fingerprints (Sub) according to the ten structural clusters, Table 1. No information about HCT116 activity was used. A trend for clustering according to structural cluster features of the compounds was observed, Figure 4.

Then, the SOM response patterns were used as a metric of similarity after normalization, where d(**x**,**n_i_**) is the Euclidian distance between the molecular descriptor vector **x** and **n***_i_* (represents the centroid vector of the *i*th SOM neuron). A threshold based on the average of SOM distance (ASD) between each molecule of the test set and all the molecules of the training set were set in accordance with the mapping of the ten structural clusters on SOM and MAE obtained in the best RF model in order to no misclassification of the structural cluster was obtained. The applicability domain of the model is defined as containing all molecules of the training set that were mapped as belonging to one of the ten clusters on SOM with an ASD lower than 0.421. Therefore, using this threshold for the test set, 873 molecules belonging to the applicability domain of the HCT116 model were obtained, with R^2^ = 0.758, RMSE = 0.681 and MAE = 0.462. However, for the molecules of the test set outside the defined applicability domain (i.e., 591 molecules) worse predictions were obtained, with R^2^ = 0.669, RMSE = 0.702 and MAE = 0.489.

Finally, the best RF model and its applicability domain were validated with a final prediction set consisting of 151 molecules not used for any task before, which were recently reported in the literature [50,51,52]. Only 50 molecules of this data set were in the applicability domain of the HCT116 model and were predicted with acceptable accuracy of R^2^ = 0.544, RMSE = 1.024 and MAE = 0.879. The SMILES strings of this data set, the corresponding experimental and predicted activities are also available as Supplementary Material.

### 3.3. Exploration of NMR Descriptors for QSAR Approach B

The NMR descriptors were generated using the following ranges: (1) 1.5 (133 descriptors), 1.0 (200 descriptors), and 0.5 ppm (400 descriptors) for ^13^C; and (2) 0.2 (61 descriptors), 0.1 (120 descriptors) and 0.05 (240 descriptors) ppm for ^1^H. Exploratory QSAR experiments employed three NMR descriptors sets (with ^1^H-NMR descriptors, ^13^C-NMR descriptors and combining ^1^H- and ^13^C-NMR descriptors) followed by RF prediction of two classes of anticancer activity (i.e., moderate-active-to-active with IC_50_ < 156 µg/mL and inactive with IC_50_ ≥ 156 µg/mL) within a OOB estimation procedure (Table 7). In Table 7, only three of the best models of the nine models, which were trained combining ^1^H- and ^13^C-NMR descriptors, are represented.

The best model was achieved using 0.1 and 0.5 ppm ranges for ^1^H and ^13^C, respectively, in total 520 NMR descriptors (bold highlighted in Table 7). For the HCT116 NMR model, the data are imbalanced as concerns the moderate-active-to-active and inactive classes (34, 76 and 9, 27 samples for the moderate-active-to-active and inactive classes of the training and test sets, respectively), and therefore in order to alleviate this problem the class weights were adjusted to 50:50, Table 8. This procedure achieved an improvement of the predictive power of the model for training and test sets.

For the best model, the results were analyzed with respect to the most active samples, i.e., with IC_50_ values lower than 5 μg/mL. In the training set, there are two samples (one crude extract, PTM-304 and one fraction, PTM-128_F8) that are predicted to be inactive i.e., FNs, with probability of being moderate-active-to-active lower than 0.25, and five samples that are correctly predicted i.e., TPs, with probability of being moderate-active-to-active higher than 0.80 (one crude extract, PTM-46 and four fractions, PTM-128_F9, PTM-29_F5, PTM-81_F2_F3, and PTM-420_F5). In relation to the test set, there are two samples with IC_50_ values lower than 5 μg/mL (one crude extract, PTM-99 and one fraction, PTM-29_F2), both are incorrectly predicted, i.e., FNs, with values of probability of being moderate-active-to-active very low (0.07 and 0.22, respectively). For pure compounds, none were in the training set because of the low amount and the fact that they are all inactive. When the five pure compounds present in the test set were predicted, the model predicted them as being moderate-active-to-active when all are inactive, with the range of probability to be moderate-active-to-active ranging from 0.57 to 0.73. This may be due to the lack of representation of these compounds in the training set that is only constituted by crude extracts and fractions.

Finally, the best RF model was validated with a final prediction set consisting of five pure compounds not used for any task before, which were recently isolated and purified in our group. These five marine natural products appear to be of the same structural family of macrocyclic compounds, however their chemical structure has not yet been fully elucidated, and therefore, are excellent candidates for this QSAR approach B model. In Table 9, we show the predictions obtained using the best model for the five pure compounds that had not previously been tested against the HCT116 cell line. For the test set, no misclassifications between moderate-active-to-active and inactive classes were obtained with probability of being moderate-active-to-active higher than 0.77 and probability of being moderate-active-to-active lower than 0.39, respectively. In this way, and with a high degree of confidence, we can only affirm that the compound PTM-99_F2_F27 belongs to the inactive class and only this one should be validated experimentally.

However, the five compounds were tested experimentally against the HCT116 cell line, and for the standard range considered in the biological assays it was difficult to calculate an IC_50_ for any of the compounds. Since the model used had a high cut-off, assays with high concentrations were carried out at the concentration of 125 μg/mL and the following absorbance results were found: 0.300, 0.324, 0.056, 0.289 and 0.076 for PTM-99_F2_F27, PTM-99_F2_F31, PTM-420_F4_F15, PTM-420_F5_F42 and PTM-420_F5_F43, respectively. Absorbance for the negative control, using the DMSO solvent, and positive control, using the reference compound 5-FU, of 0.322 and 0.052, respectively, were obtained.

#### Analysis of NMR Descriptors Identified as Relevant for Modeling HCT116 Activity in the Best RF Model

The fifty most relevant descriptors, found by the RF algorithm using the MeanDecreaseAccuracy parameter (Mean Decrease in Accuracy) [53] were analyzed and represented in Table 10.

Interestingly, there are nine descriptors that codify ^1^H-NMR data out of the ten most important NMR descriptors. From those nine ^1^H-NMR descriptors, six descriptors codify saturated alkyl groups (14, 2, 3, 4, 5 and 6), where five out of the six descriptors are correlated to the methyl group. The other three ^1^H-NMR descriptors codify the bonding to an electronegative atom such as N, O or halogen (44 and 45) and vinyl groups (52). The only ^13^C-NMR descriptor in the ten most important NMR descriptors discriminated the alcohol, ether or alkyne functional groups (271). Moreover, we also verified that the importance by activity classes (moderate-active-to-active and inactive classes) for each of the ten most important descriptors is more or less similar, which seems to indicate that although they are the most relevant descriptors, they do not allow the discrimination among the classes of activity. On the other hand, the analysis of the fifty most important descriptors permitted the identification of seven descriptors (H: 32, 51, 73, 13; C: 170, 352, 280) that allow the discrimination among the classes of activity. The descriptors that give the moderate-active-to-active class a higher weight are C (352) and H (13), which encode aromatic, olefinic or nitrile carbon atoms and saturated alkyl methylene proton atoms, respectively.

## 4. Conclusions

The results suggest that the chemoinformatics QSAR approach relying on a ligand-based methodology either based on the molecular structures or the NMR spectra, corroborated with an experimental approach and could be used to predict new compounds inhibitors against the human colon carcinoma HCT116 cell line. To our knowledge, the QSAR regression model developed here, Approach A, is the largest study ever performed with regard both to the number of compounds involved and to the number of structural families involved in the modeling of inhibitory activity against HCT116 [10,11,12,13,14,15,16,17,18,19,20,21,22]. The performance achieved by the NMR QSAR classification model, Approach B, allowed us to conclude that it was an excellent effort and a useful tool for dereplication to be developed if this study is extended to a high number of samples containing crude extracts, fractions and mainly pure compounds. This will be an interesting approach in future work. The two approaches developed (A, through molecular structures, and B, through NMR spectra) allow the development of a complementary strategy to predict new anticancer compounds against the human colon carcinoma HCT116 cell line. Approach B allows the prioritization of the isolation, purification and structural elucidation of crude extracts, fractions and pure compounds. Pure compounds that are elucidated may be subjected to model A and the compounds predicted to be most active against HCT116 line may be evaluated experimentally.

## Figures and Tables

**Table 1:** Structural Clusters and pIC_50_ Values for HCT116 within the Clusters.

Clusters ^1^	Training Set ^2^	Test Set ^2^	Average/Maximum pIC_50_^3^
I—ChEMBL1078221 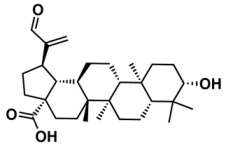	650	129	5.24/11.00
II—ChEMBL1078389 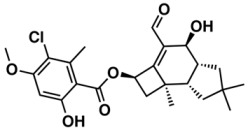	569	135	5.43/9.52
III—ChEMBL148968 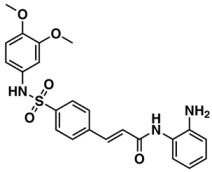	438	105	5.42/9.60
IV—ChEMBL116081 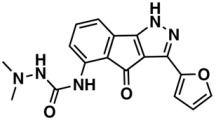	713	200	5.83/11.51
V—ChEMBL1083086 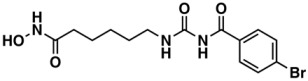	885	208	5.32/9.26
VI—ChEMBL104408 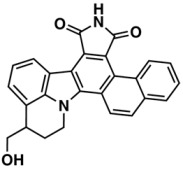	405	119	5.80/9.31
VII—ChEMBL1090871 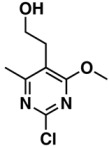	661	159	5.39/12.00
VIII—ChEMBL116614 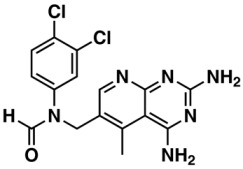	626	152	5.76/9.24
IX—ChEMBL1078573 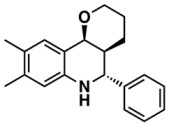	513	125	5.74/10.35
X—ChEMBL1830679 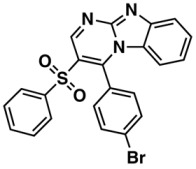	415	132	5.77/9.05

^1^ Cluster number and chemical structure of the cluster centroid; ^2^ Number of molecules; ^3^ Within the cluster for the training set.

**Table 2:** Actinomycetes genera and correspondent IC_50_ values for HCT116.

Actinomycetes Genera	Set (Number/Sample Types)	Activity Class/Average IC_50_^1^
*Actinomadura*	Tr ^2^ set (2, crude extracts)	inactive/≥156
*Micromonospora*	Tr ^2^ set (4, 1 crude extract and 3 fractions)	active/33.95
*Micromonospora*	Tr ^2^ set (11, 3 crude extracts and 8 fractions)	inactive/≥156
*Salinispora*	Tr ^2^ set (1, 1 fraction)	active/9.8
*Salinispora*	Tr ^2^ set (20, 9 crude extracts and 11 fractions)	inactive/≥156
*Streptomyces*	Tr ^2^ set (20, 11 crude extracts and 9 fractions)	active/16.26
*Streptomyces*	Tr ^2^ set (16, 9 crude extracts and 7 fractions)	inactive/≥15
*Actinomadura*	Te ^3^ set (1, crude extract)	inactive/≥156
*Brevibacterium*	Te ^3^ set (1, crude extract)	inactive/≥156
*Micromonospora*	Te ^3^ set (1, crude extract)	active/7.9
*Micromonospora*	Te ^3^ set (11, 1 crude extract, 5 fractions, and 5 pure compounds)	inactive/≥156
*Salinispora*	Te ^3^ set (1, crude fraction)	active/4.94
*Salinispora*	Te ^3^ set (7, 5 crude extracts and 2 fractions)	inactive/≥156
*Streptomyces*	Te ^3^ set (7, 2 crude extracts and 5 fractions)	active/26.31
*Streptomyces*	Te ^3^ set (7, 3 crude extracts and 4 fractions)	inactive/≥156

^1^ μg/mL; ^2^ Training set; ^3^ Test set.

**Table 3:** Exploration of two collections of empirical descriptors for the quantitative structure-activity relationship *k*-nearest neighbors (QSAR *k*-NN) model of pIC_50_ for the training set with a ten-fold cross-validation. The best models are highlighted in bold.

Descriptors (#)	CFS Search Type	N_O_. of Selected Descriptors	R^2^	RMSE	MAE	% error ≥ 1/% error < 1 ^1^
E-State (79) ^2^	GSW ^4^	13	0.174	1.208	0.927	38/62
MACCS (166) ^2^	PSOs ^5^	34	0.512	0.937	0.665	22/78
Sub (307) ^2^	PSOs ^5^	63	0.372	1.055	0.797	30/70
SubC (307) ^2^	BF ^6^	63	0.509	0.942	0.671	23/77
AP2D (780) ^2^	PSOs ^5^	120	0.442	1.007	0.702	23/77
APC2D (780) ^2^	PSOs ^5^	174	0.589	0.866	0.589	18/82
PubChem (881) ^2^	PSOs ^5^	252	**0.696**	**0.742**	**0.500**	**14/86**
CDK (1024) ^2^	PSOs ^5^	283	**0.725**	**0.707**	**0.474**	**12/88**
CDK Ext (1024) ^2^	PSOs ^5^	257	**0.718**	**0.717**	**0.476**	**13/87**
CDK graph (1024) ^2^	PSOs ^5^	179	0.644	0.807	0.546	16/84
KR (4860) ^2^	PSOs ^5^	192	0.604	0.847	0.591	19/81
KRC (4860) ^2^	PSOs ^5^	160	0.618	0.832	0.579	18/82
1D2D (1438) ^3^	PSOs ^5^	416	**0.703**	**0.737**	**0.493**	**13/87**
1D2D3D (1869) ^3^	PSOs ^5^	489	**0.705**	**0.733**	**0.493**	**13/87**

^1^ Percent of molecules predicted with absolute error above or below 1; ^2^ Fingerprints; ^3^ Molecular descriptors; ^4^ GreedyStepwise option for search; ^5^ PSOsearch option for search; ^6^ BestFirst option for search. Abbreviations: RMSE, root mean square error; MAE, mean absolute error.

**Table 4:** Performance of different machine learning algorithms. The best models are highlighted in bold.

Models		ML		
		RF ^1^	SVM ^2^	*K*-NN ^2^
1D2D ^3^	R^2^	0.730	0.647	0.703
	RMSE	0.708	0.800	0.737
	MAE	0.523	0.566	0.493
	% error ≥ 1/% error < 1 ^7^	13/87	16/84	13/87
1D2D3D ^4^	R^2^	0.729	0.615	0.705
	RMSE	0.713	0.842	0.733
	MAE	0.525	0.572	0.493
	% error ≥ 1/% error < 1 ^7^	13/87	17/83	13/87
PubChem ^5^	R^2^	**0.751**	0.677	0.696
	RMSE	**0.664**	0.762	0.742
	MAE	**0.466**	0.535	0.500
	% error ≥ 1/% error < 1 ^7^	**12/88**	15/85	14/86
CDK ^6^	R^2^	0.753	**0.744**	**0.725**
	RMSE	0.665	**0.674**	**0.707**
	MAE	0.471	**0.469**	**0.474**
	% error ≥ 1/% error < 1 ^7^	11/89	**12/88**	**12/88**

^1^ Out-of-bag (OOB) estimation for the training set; ^2^ Ten-fold cross-validation for the training set; ^3^ 1438 and 416 descriptors for random forest (RF) and support vector machines/*k*-nearest neighbors (SVM/*k*-NN), respectively; ^4^ 1869 and 489 descriptors for RF and SVM/*k*-NN, respectively; ^5^ 881 and 252 descriptors for RF and SVM/*k*-NN, respectively; ^6^ 1024 and 257 descriptors for RF and SVM/*k*-NN, respectively; ^7^ Percent of molecules predicted with absolute error above or below 1.

**Table 5:** The predictions of the best HCT116 QSAR model by the ten structural clusters for training and test sets. The best models are highlighted in bold.

Clusters	Training Set				Test Set			
	#	R^2^	RMSE	MAE	#	R^2^	RMSE	MAE
**I**	650	0.702	**0.721**	0.479	129	0.613	0.673	0.455
**II**	569	0.766	0.627	0.446	135	0.781	0.619	0.461
**III**	438	0.792	0.648	0.459	105	0.697	0.737	0.516
**IV**	713	0.759	**0.703**	0.459	200	0.682	0.821	0.533
**V**	885	0.685	0.658	0.481	208	0.658	0.734	0.489
**VI**	405	0.649	0.646	0.460	119	0.637	0.616	0.462
**VII**	661	0.790	0.652	0.445	159	0.776	0.625	0.430
**VIII**	626	0.636	**0.708**	0.512	152	0.706	0.585	0.432
**IX**	513	0.846	0.599	0.412	125	0.794	0.720	0.487
**X**	415	0.746	0.628	0.440	132	0.767	0.659	0.448

**Table 6:** Analysis of descriptor importance using to build the best QSAR model for the prediction of the pIC_50_ against HCT116.

Code	DI ^1^	Chemical Pattern
HEC_2	17th	≥16C
HEC_19	16th	≥2O
ESSSR_157	10th	≥3 any ring size 5
ESSSR_261	5th	≥4 aromatic rings
SAP_301	18th	N-O
SAP_305	19th	N-S
SANN_335	7th	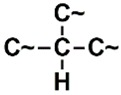 ~Any bond order but no aromatic bond
SANN_338	4th	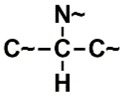 ~Any bond order but no aromatic bond
SANN_339	11th	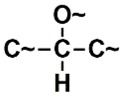 ~Any bond order but no aromatic bond
SANN_346	15th	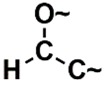 ~Any bond order but no aromatic bond
DANh_432	8th	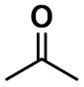
SSP_514	12th	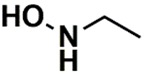
SSP_518	6th	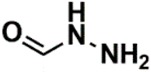
SSP_615	13th	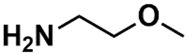
SSP_631	20th	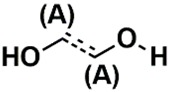
SSP_643	3rd	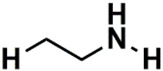
SSP_672	14th	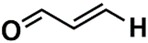
CSP_713	1st	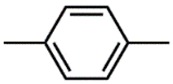
CSP_755	9th	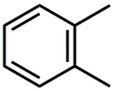
CSP_819	2nd	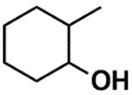

^1^ Descriptors importance.

**Table 7:** Exploration of three collections of NMR descriptors for the QSAR RF model of HCT116 activity classes for the training and test sets. The best models are highlighted in bold.

	Model	# ^2^	Training ^1^/Test Sets							
			TP ^3^	TN ^4^	FP ^5^	FN ^6^	SE ^7^	SP ^8^	Q ^9^	G-Mean ^10^
**^13^C**	0.5	400	12/3	38/20	11/7	13/6	0.48/0.33	0.78/0.74	0.68/0.64	0.61/0.50
	1	200	13/3	35/16	8/7	12/6	0.52/0.33	0.81/0.70	0.71/0.59	0.65/0.48
	1.5	133	12/2	42/20	7/7	13/7	0.48/0.22	0.86/0.74	0.73/0.61	0.64/0.41
**^1^H**	0.05	240	13/2	41/20	8/7	12/7	0.52/0.22	0.84/0.74	0.73/0.61	0.66/0.41
	0.1	120	15/2	40/19	9/8	10/7	0.60/0.22	0.82/0.70	0.74/0.58	0.70/0.40
	0.2	61	14/2	39/18	10/9	11/7	0.56/0.22	0.80/0.67	0.72/0.56	0.67/0.39
**^1^H and ^13^C**	0.05; 0.5	640	13/4	44/21	5/6	12/5	0.52/0.44	0.90/0.78	0.77/0.69	0.68/0.59
	0.1; 0.5	520	14/5	44/19	5/8	11/4	0.56/0.56	0.90/0.70	0.78/0.67	**0.71/0.63**
	0.1; 1	320	13/3	44/19	5/8	12/6	0.52/0.33	0.90/0.70	0.77/0.61	0.68/0.48

^1^ OOB estimation; ^2^ Number of descriptors; ^3^ True positives; ^4^ True negatives; ^5^ False positives; ^6^ False negatives; ^7^ Sensitivity, the ratio of true positives to the sum of true positives and false negatives; ^8^ Specificity, the ratio of true negatives to the sum of true negatives and false positives; ^9^ Overall predictive accuracy, the ratio of the sum of true positives and true negatives to the sum of true positives, true negatives, false positives and false negatives; ^10^ The square root of the product of sensitivity and specificity.

**Table 8:** Balance the moderate-active-to-active and inactive classes for the best NMR RF model of HCT116 activity classes for the Training and Test Sets.

Sets	TP ^1^	TN ^2^	FP ^3^	FN ^4^	SE ^5^	SP ^6^	Q ^7^	G-Mean ^8^
Training	18	36	13	7	0.72	0.74	0.73	0.73
Test	6	17	10	3	0.67	0.63	0.64	0.65

^1^ True positives; ^2^ True negatives; ^3^ False positives; ^4^ False negatives; ^5^ Sensitivity, the ratio of true positives to the sum of true positives and false negatives; ^6^ Specificity, the ratio of true negatives to the sum of true negatives and false positives; ^7^ Overall predictive accuracy, the ratio of the sum of true positives and true negatives to the sum of true positives, true negatives, false positives and false negatives; ^8^ The square root of the product of sensitivity and specificity.

**Table 9:** Prediction of activity classes against HCT116 of the five pure compounds with the best model.

Code	Activity Class	Probability of Being Moderate-Active-to-Active
PTM-99_F2_F27	Inactive	0.26
PTM-99_F2_F31	Inactive	0.42
PTM-420_F4_F15	Moderate-active-to-active	0.64
PTM-420_F5_F42	Moderate-active-to-active	0.53
PTM-420_F5_F43	Moderate-active-to-active	0.55

**Table 10:** Analysis of NMR Descriptors for modeling HCT116 activity in the best RF model.

H or C (# ^1^)	NMR Range (ppm)	DI ^2^	Importance for Classes		Pattern Identification
			MAct-Act ^3^	InAct ^4^	
H (14)	1.3019–1.4019	1st	5.43	5.97	Saturated 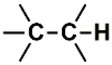
H (44)	4.3019–4.4019	2nd	5.90	4.46	Z = O, N, X ^5^ 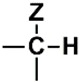
H (2)	0.1019–0.2019	3rd	6.43	4.01	Saturated 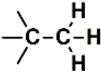
H (3)	0.2019–0.3019	4th	4.79	4.20	Saturated 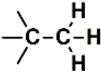
H (4)	0.3019–0.4019	5th	3.94	4.60	Saturated 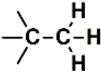
H (45)	4.4019–4.5019	6th	4.49	4.13	Z = O, N, X ^5^ 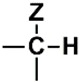
H (5)	0.4019–0.5019	7th	3.27	4.04	Saturated 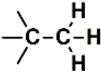
C (271)	74.9927–75.4927	8th	2.00	2.98	Alcohol and ethers 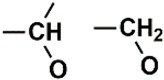 Alkynes 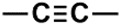
H (6)	0.5019–0.6019	9th	1.77	3.25	Saturated 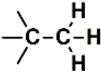
H (52)	5.1019–5.2019	10th	1.87	2.67	Vinylic 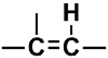
H (32)	3.1019–3.2019	12th	0.881	2.87	Z = O, N, X ^5^ 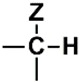
H (51)	5.0019–5.1019	15th	0.0887	2.73	Vinylic 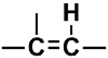
C (170)	24.4927–24.9927	20th	0.712	2.14	Allylic 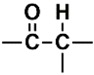 *N*-Alkyl amines 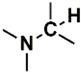 Saturated 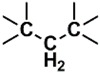
C (352)	115.4927–115.9927	21th	2.12	0.833	Aromatic 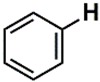 Olefinic 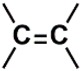 Nitrile 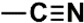
C (280)	79.4927–79.9927	26th	0.0743	1.88	Alcohol and ethers 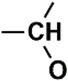 Alkynes 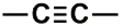
H (73)	7.2019–7.3019	32th	0.083	1.91	Aromatic 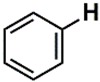 Conjugated olefinic 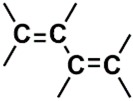
H (13)	1.2019–1.3019	49th	1.42	0.0695	Saturated 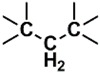

^1^ Number of descriptor; ^2^ Descriptors importance; ^3^ Moderate-active-to-active class; ^4^ Inactive class; ^5^ Halogen.

## Supplementary Materials

The following are available online at http://www.mdpi.com/2218-273X/8/3/56/s1, Tables S1–S3: the SMILES strings of the training, test, and test 2 sets, and the corresponding experimental and predicted activities against HCT116 for Approach A, respectively; Tables S4–S6: the code and type and the actinomycete genus of the samples comprising the training, test, and test 2 sets and the corresponding experimental and predicted activity classes against HCT116 for Approach B, respectively.
